# Estimating average annual per cent change in trend analysis

**DOI:** 10.1002/sim.3733

**Published:** 2009-10-23

**Authors:** Limin X Clegg, Benjamin F Hankey, Ram Tiwari, Eric J Feuer, Brenda K Edwards

**Affiliations:** 1 Office of Inspector General, U.S. Department of Veterans AffairsWashington, DC, U.S.A.; 2 Information Management Services, Inc.Silver Spring, MD, U.S.A.; 3 Office of Biostatistics, Center for Drug Evaluation and Research, U.S. Food and Drug AdministrationU.S.A.; 4 Surveillance Research Program, National Cancer InstituteBethesda, MD, U.S.A.

**Keywords:** confidence interval for trends, geometric means, trend comparisons

## Abstract

Trends in incidence or mortality rates over a specified time interval are usually described by the conventional annual per cent change (cAPC), under the assumption of a constant rate of change. When this assumption does not hold over the entire time interval, the trend may be characterized using the annual per cent changes from segmented analysis (sAPCs). This approach assumes that the change in rates is constant over each time partition defined by the transition points, but varies among different time partitions. Different groups (e.g. racial subgroups), however, may have different transition points and thus different time partitions over which they have constant rates of change, making comparison of sAPCs problematic across groups over a common time interval of interest (e.g. the past 10 years). We propose a new measure, the average annual per cent change (AAPC), which uses sAPCs to summarize and compare trends for a specific time period. The advantage of the proposed AAPC is that it takes into account the trend transitions, whereas cAPC does not and can lead to erroneous conclusions. In addition, when the trend is constant over the entire time interval of interest, the AAPC has the advantage of reducing to both cAPC and sAPC. Moreover, because the estimated AAPC is based on the segmented analysis over the entire data series, any selected subinterval within a single time partition will yield the same AAPC estimate—that is it will be equal to the estimated sAPC for that time partition. The cAPC, however, is re-estimated using data only from that selected subinterval; thus, its estimate may be sensitive to the subinterval selected. The AAPC estimation has been incorporated into the segmented regression (free) software Joinpoint, which is used by many registries throughout the world for characterizing trends in cancer rates. Copyright © 2009 John Wiley & Sons, Ltd.

## 1. INTRODUCTION

Studies of disease incidence and mortality rates over time and across demographic subgroups play an important role in guiding national programs for disease prevention, control, and surveillance. For example, the *Annual Report to the Nation on the Status of Cancer* provides updated information on cancer incidence and mortality trends in the United States. It is published collaboratively by the American Cancer Society, the Centers for Disease Control and Prevention, the National Cancer Institute (NCI), and the North American Association of Central Cancer Registries. This annual report provides trends in age-adjusted incidence and mortality rates for the top 15 cancers, both long-term and short-term, by sex and race [1].

One popular method of trend analysis is to estimate the conventional annual per cent change (cAPC) for age-adjusted rates [2, 3]. The cAPC is estimated by fitting a simple linear model: the logarithm of the yearly age-adjusted rates first is regressed on time, then a transformation of the slope is used to calculate the per cent change per year. The cAPC is easy to calculate and interpret. For long-term trend analysis, however, the linearity of rates on the logarithmic scale, implying a constant rate of change, may not apply over the entire time period of interest.

When the trend is not constant over the entire time period of interest, the nonlinearity of the trend may be characterized using the annual per cent change from segmented analysis (sAPC). This approach assumes that the change in age-adjusted rates is constant over each time partition defined by the transition points, but varies among different time partitions [1,2]. When comparing trends for different groups (such as racial subgroups), different groups may have different transition points and thus different time partitions over which they have constant rates of change; the comparison of group sAPCs is problematic over a common time interval of interest (e.g. the past 5 or 10 years).

**Figure 1:**
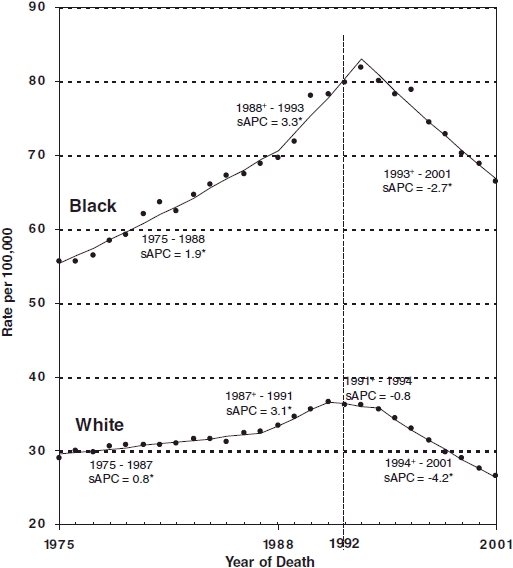
U.S. Mortality Rates, Prostate, 1975.2001, By Race. Source: National Center for Health Statistics public use data file for the total US. Rates are age-adjusted to the 2000 US standard million population by 5-year age groups. *The sAPC (annual per cent change from segmented analysis) is significantly different from zero (*p*<0.05).

For example, the segmented regression analysis for age-adjusted mortality rates for prostate cancer in the U.S. from 1975 to 2001 (Figure 1, based on data from the National Center for Health Statistics, NCHS 2004 [4]) consists of four line segments for whites (1975–1987, 1987+–1991, 1991+–1994, and 1994+–2001) and three line segments for blacks (1975–1988, 1988+–1993, and 1993+–2001). Note that we define *t*^+^ = *t*+Δ*t* with Δ*t* → 0 when *t* is continuous and Δ*t* = 1 when *t* is discrete. Because of the difference in the last transition points for whites and blacks over the time period 1975–2001, the time period of the most current trend is from 1994+ to 2001 for whites, but from 1993+ to 2001 for blacks. Because the time periods of most current trends are different, the estimated most current trends for whites (decreasing 4.2 per cent annually) and for blacks (decreasing 2.7 per cent annually) are not directly comparable. Moreover, the introduction of prostate-specific antigen (PSA) screening and new treatments (most notably the use of androgen-deprivation therapy in the adjuvant setting) over the last 10 years raises the question of possible racial disparity in the annual per cent decline over this period. The sAPCs from the segmented regression analysis, however, do not allow direct comparison between blacks and whites over the 10-year time period from 1992 to 2001 because the sAPC for whites was –0.8 per cent for 1991–1994 and –4.2 per cent for 1994+–2001, while for blacks it was 3.3 per cent from 1988 to 1993 and –0.7 per cent from 1993+ to 2001.

Hence, it is essential to develop a summary measure of trends that takes into account trend transition over a common subtime period—for example the 10 years from 1992 to 2001—for the trend comparison. In addition, a summary measure that applies over the entire time period also is needed so that the overall 1975–2001 trends for whites and blacks can be compared after accounting for different racial trend transitions.

Motivated by the need for a trend analysis summary measure to facilitate trend comparisons, we propose the average annual per cent change (AAPC) to summarize and compare rates of change that are not constant over a given time period. The AAPC reduces to both the cAPC and the sAPC if the rate of change is constant over the entire time period of interest. The remainder of this paper is organized as follows: Section 2 presents the proposed AAPC. In Section 3, we apply the AAPC estimator to data from the Surveillance, Epidemiology, and End Results (SEER) Program at the NCI (see [5] for more information on the SEER Program) and compare the proposed AAPC with the cAPC. The results and the proposed methodology are discussed further in Section 4.

## 2. AVERAGE ANNUAL PER CENT CHANGE (AAPC)

### 2.1. Conventional annual per cent change (cAPC) and its estimator

Denote the observed rates at time *t_i_* as *r_i_* with the associated random variable *R_i_*, and denote the corresponding expected rate as *y_i_*= *E*(*R_i_*\*t_i_*), where the *n* observed ordered time points are *a* = *t*_1_<*t*_2_< … <*t_n_* = *b*. Assume that *t_i_* represents years and is equally spaced over [*a,b*] with *t_j+1_* = *t_j_* + 1. Also, assume that log(*γi*) is linear over the entire time interval [*a,b*], that is


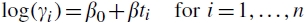
(1)

Then the annual rate of change is


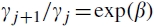


and the cAPC is


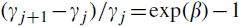


Denote 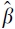 as the estimated slope of the regression line (1) based on the data (*t_i_, r_i_*), *i* = 1, …, *n*. Then, the estimated cAPC is 
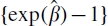
 Note that 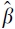 may be estimated by the approach of weighted least squares, with weights as the reciprocals of variances of age-adjusted rates, *R_i_*, to take into account the variability in the rates *R_i_*.

Note that the rates can take on only positive values, but their logarithms can have unrestricted range, and hence the normal approximation can be used to estimate the parameters. It is, therefore, appropriate to first calculate a range for log rates, and then to convert this back to a range for rates [6].

### 2.2. Segmented regression model

Suppose that the log(*γ_i_*) is nonlinear over the entire time interval [*a,b*] and that it follows the segmented linear regression model, that is


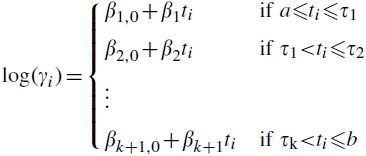
(2)

In this model, the time interval [*a,b*] is partitioned into *k* + 1 segments by the *k* transition points *τ_j_, j* = 1, …, *k*, with *a* = *t*_1_ < … <*t*_*n*1_ ≤τ1 <*t*_*n*1_ + 1< … <*t*_*N*__*j*_≤τ_*j*_<*t*_*j*__*j*_ + 1 < … <*t*_*N*__*k*+1_ = *t*_*n*_ = *b*, where 
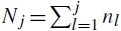
 and *n _j_* represents the number of observed data points between the transition points τ_*j* – 1_ and τ_*j*_, that is in the time interval (*τ*_*j*–1_, *τ_j_*], with 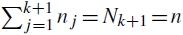. For the purpose of notational convenience, we define τ0 = *a*¯ and τ _*k* + 1_ = *t*_*n*__*k*+1_ = *t*_*n*_ = *b*, where *a*¯ = *a* – Δ*t* with Δ*t* → 0 when *t_i_* is continuous and Δ*t* = 1 when *t_i_* is discrete. The segmented regression model (2) indicates that the rate of change is constant within each of the (*k* + 1) partitions: [*a*, τ_1_], (τ_1_, τ_2_], …, (τ*k*–1, τ*k*], and (τ*k*, *t_n_*], with the constant rate of change corresponding to the partition (τ _*j*–1_, τ_*j*_] being {exp(β_*j*_) – 1}, *j* = 1,…, *k* + 1. We can rewrite equation (2) as


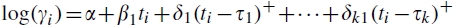


where


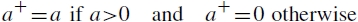


with the constraints:


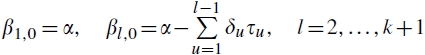



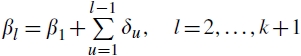


These constraints guarantee the continuity of (2) at τ points.

### 2.3. Proposed summary measure AAPC

Assume that log(*γ_i_*) is nonlinear over the time interval [*a,b*] and follows the segmented regression model (2). We propose to summarize the (*k* + 1) change rates in the entire time interval [*a,b*] with the AAPC, conditional on the transitional points τ _*j*_, *j* = 1, …, *k*. The AAPC is defined as

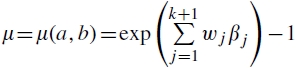
(3)
where the normalized (i.e. 
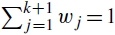
) weights *w_j_* = (*τ_j_* – *τ*_*j*–_)/(*b*–*a*¯), *j* = 1,…, *k* + 1, with τ_0_ = *a*¯ and τ_*k* + 1_ = *b* as defined earlier. When the *t_i_* are equally spaced, the weights reduce to *w_j_* = *n_j_*/*n*, with *n _j_* representing the number of observed data points between the transition points *τ*_*j*–1_ and *τ*_*j*_, (*τ*_*j*–1_, *τ*_*j*_]. Note that the weights *w_j_* are proportional to the corresponding lengths of the time partitions, and they are proportional to the numbers of data points within the partition (*τ* _*j*–1_, *τ*_*j*_], *j* = 1, …, *k* + 1, when the *t_i_* are equally spaced.

When the rate of change is constant over the entire time period [*a,b*], it is clear that the AAPC in equation (3) reduces to both the cAPC that is commonly used and the sAPC from the segment analysis; otherwise, the AAPC is the geometric mean of the annual changes from all of the partitions. More specifically, from (3)


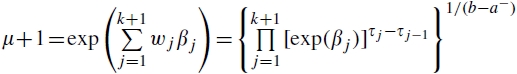


When *t_i_* are equally spaced, this reduces to


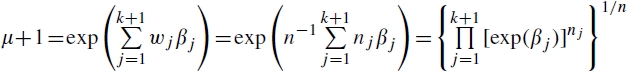


The fact that (μ + 1) is the geometric mean of the annual changes exp(β_*j*_) or, equivalently, log (μ+1) is the arithmetic mean of log(annual change), β_*j*_, from all the partitions, motivates us to name the summary measure μ the AAPC. Thus, the AAPC is obtained easily if we know the sAPC in each of the *k* + 1 partitions. For example, if we know that the three sAPC values in [*a,b*] are 10 per cent, –3 per cent, and 2 per cent (i.e. *k* = 2) with equal weighting (say, each line segment has six data points, i.e. *n _j_* = 6) and the *t_i_* are equally spaced, then the AAPC over the 18-year time period [*a,b*] is given by 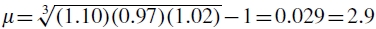 per cent. This means that the age-adjusted rates increased 2.9 per cent annually on average during the 18-year period of time.

Denote the estimator of β_*j*_ by 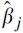. The AAPC μ over the period [*a,b*] is estimated by


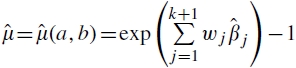
(4)

Using the delta method, a general approach for variance estimation of functions of random variables [7, 8], we estimate the standard error of 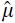 by


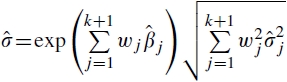
(5)

where 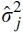 is the variance estimator of 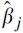. Note that, as mentioned earlier, the 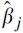 may be taken to be the weighted least-squares estimators to take into account the variability in *R_i_*.

Statistical significance tests pertaining to the departure of the AAPC from zero or comparison of AAPCs between groups for a given time period can be performed easily on the log scale. To construct a confidence interval (CI) for μ or to compare μ from different groups, the distribution of 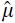 is needed. Under the standard assumption that the counts for the age-adjusted rates are Poisson and the person-years in the denominator are fixed constants [6], for large counts the age-adjusted rates on the log scale, that is log(*R_i_*), are asymptotically normally distributed. Consequently, 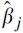 and log 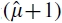 as well, are asymptotically normal. Hence, the CIs for the AAPC can be calculated first on the log scale, under the asymptotic normality of and then transformed back through exponentiation. More specifically, the 100(1 – α) per cent lower (μ_L_) and upper (μ_U_) confidence limits for the AAPC, μ, are given by


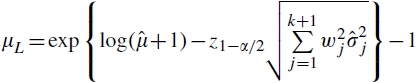


and


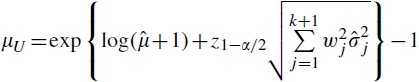
(6)

where *z*_α_ is the αth quantile of the standard normal distribution, μ is defined as in equation (4) and 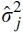 is the variance estimator of 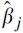 as in equation (5). If the CI contains zero, then there is no evidence to reject the null hypothesis *H*_0_: μ = 0 at the significance level of α; otherwise, we reject *H*_0_ in favor of *H*_1_: μ≠0 and conclude that the rate of change is increasing on average over the time interval [*a,b*] if the lower confidence limit μ_L_ is positive, or that the rate of change is decreasing if the upper confidence limit μ_U_ is negative. If *p*-values are preferred, rather than the 95 per cent CIs, the computation of *p*-values is straightforward under the asymptotic normality.

To compare AAPCs from two different groups, we may employ a ratio statistic (μ_1_ + 1)/(μ_2_ + 1) where the subscripts 1 and 2 indicate Group 1 and Group 2, respectively. The 100(1 – α) per cent confidence limits for the ratio (μ_1_ + 1)/(μ_2_ + 1) are


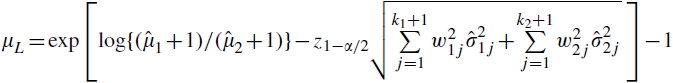


and


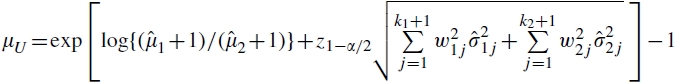
(7)

Note that, if the CI contains unity, there is no evidence to reject the null hypothesis *H*_0_: (μ_1_ + 1)/(μ_2_ + 1) = 1 at the significance level of α otherwise, we reject *H*_0_ in favor of *H*_1_: (μ_1_ + 1)/(μ_2_ + 1) ≠ 1 and conclude that, on average, the annual rate of change for Group 1 is more rapid than for Group 2 over the time interval [*a,b*] if μ_L_ > 1 or, alternatively, the rate of annual change for Group 1 is slower than for Group 2 if μ_U_ <1.

### 2.4. AAPC for subtime intervals of specified length

In addition to summarizing and comparing the (long-term) trend for the entire time period of [*a,b*] during which the data are observed, the AAPC also may be used to summarize any short-term trend for any subinterval [*s*_1_, *s*_2_] within [*a,b*], where *a*≤*s*_1_ <*s*_2_ ≤*b*. Based on equation (3), the AAPC in the subinterval [*s*_1_, *s*_2_], denoted as μ(*s*_1_, *s*_2_), is


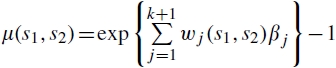
(8)

and the AAPC μ(*s*_1_,*s*_2_) is estimated by


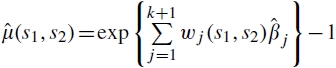
(9)

where *w_j_* (*s*_1_, *s*_2_) are the normalized weights over [*s*_1_, *s*_2_], denoting the proportion of the length (*s*_2_-*s*_1_) that falls within the partitioning intervals (*τ*_*j*–1_, *τ*_*j*_], *j* = 1, …, *k* + 1. Specifically,


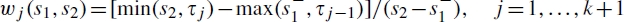


Note that we use the notation *τ*_0_ = α¯ and *τ*_*k* + 1_ = *b* in *w_j_* (*s_1_*, *s*_2_).

When *t*_i_ is discrete and equally spaced, the weights reduce to


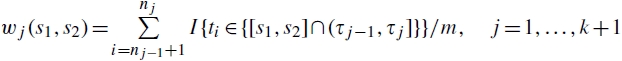


and


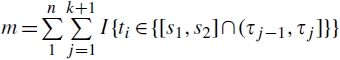


where we define *n*_0_ = 0, and *I*{*c*} is the indicator variable, with *I*{*c*} = 0 otherwise. Thus, *m* is the total number of data points covered by [*s*_1_, *s*_2_], and *w_j_* (*s*_1_, *s*_2_) is the proportion of data points (out of a total of *m*) covered in the *j*th partition (*τ*_*j*–1_, *τ*_*j*_], *j* = 1, …, *k* + 1, respectively.

Therefore, after all *β_j_* in the segment regression model (2) are estimated, we can easily use these estimates 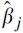 to calculate μ(*s*_1_, *s*_2_) for any subinterval [*s*_1_, *s*_2_]. The confidence limits in equations (6) and (7) naturally extend to μ(*s*_1_, *s*_2_) as well, by substituting *w_j_* (*s*_1_, *s*_2_) for *w_j_*. In addition, μ(*s*_1_, *s*_2_) clearly is the geometric mean of the annual changes for all of the partitions within [*s*_1_, *s*_2_].

The U.S. federal government publications in cancer statistics typically report and compare the last 10- (and 5-) year trends (in addition to long-term trends using all available data) in assessing current progress on cancer control. μ(*s*_1_, *s*_2_) is especially valuable when comparing trends of different population groups over a subinterval [*s*_1_, *s*_2_] of [*a, b*]. For example, it may be desirable to compare the 10-year (say, from 1993 to 2002) trends of cancer incidence rates for blacks and whites. For these groups, the segmented regression models over [*a, b*] are likely to have different partitions between blacks and whites in the 10-year period defined by their group-specific transition points; that is the number of partitions could differ (e.g. with two and three different line segments for blacks and whites, respectively), and/or different transition points for partitions could differ (e.g. the transition point being 1995 for blacks but 1994 for whites). Denote the slope of the *j*th line segment as 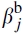 for blacks and 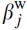 for whites. Suppose that, during the 10 years from 1993 to 2002, the only transition point is 1995 for blacks and that the transition points are 1994 and 1998 for whites; then 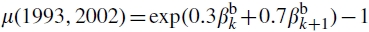 for blacks and exp 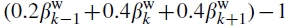 for whites. Similarly, if the only transition point is at 1993 for blacks but at 1996 for whites then 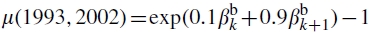
for blacks and exp 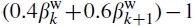
for whites. Note that the value of k may differ for blacks and whites as well.

## 3. EXAMPLES

As an illustration, the sex-specific age-adjusted cancer incidence rates and trends, including the cAPC, sAPC, and AAPC, for the top 15 cancers, were calculated using 1975-2002 population-based cancer registry data (SEER-9) collected by the SEER Program at the NCI. Since 1973, the SEER Program has collected data on all primary cancers occurring in residents of defined geographic regions. The Program currently collects and publishes cancer incidence and survival data covering approximately 26 per cent of the U.S. population (see www.seer.cancer.gov for detailed information).

These yearly age-adjusted cancer incidence rates also are adjusted for reporting delays (see [9,10] for more details) and standardized to the 2000 U.S. Standard Population (Census pp. 25–1130). The segmented regression model of equation (2) was fitted using the default setting of weighted least squares in Version 3.0 of the Joinpoint software developed by the NCI (see http://srab.cancer.gov/joinpoint and [11–13] for details).

The cAPC was calculated assuming that there were no transition points in the given time interval of interest, and the AAPC was calculated based on equation (4), using the results from the segmented regressions over the time period 1975-2002. Both the cAPC and AAPC also were calculated for the last 10 years, 1993–2002. Note that the AAPC, μ(1993, 2002), was estimated based on equation (9), using the results from the same segmented regression analysis over the period 1975–2002, whereas the cAPC was re-estimated based only on the 1993–2002 data.

Table I represents sex-specific results of estimated sAPCs for each of the line segments from the segmented regression analyses, the estimated cAPCs, and AAPCs for the entire time period (1975–2002) and for the last 10 years (1993–2002), and their 95 per cent CIs as well. The trends of these top 15 cancer sites cover from 0 to 3 transition points.

**Table I:** Estimated annual per cent changes of cancer incidence rates from the segmented regression analyses (sAPC) and the conventional method (cAPC), and the proposed new method (AAPC) with 95 per cent confidence intervals for the top 15 cancer sites: 1975–2002.

	Segmented regression analyses (1975–2002)‡																			
	Trend 1		Trend 2		Trend 3		Trend 4		1975–2002						1993–2002					
	Year	sAPC§ (percnt)	Year	sAPC§ (percnt)	Year	sAPC§ (percnt)	Year	sAPC§ (percnt)	cAPC§ (percnt)	95 percnt	C.I	AAPC§ (percnt)	95 percnt	C.I	cAPC§ (percnt)	95 percnt	C.I	AAPC§ (percnt)	95 percnt	C.I
All Sites∥																				
Both Sexes	1975–1983	0.9∥	1983+–1992	1.8∥	1992+–1995	-1.7	1995+–2002	0.3	0.8∥	0.6	1.0	0.8∥	0.4	1.1	0.1	-0.2	0.3	-0.3	-1.1	0.5
Male	1975–1989	1.3∥	1989+–1992	5.2∥	1992+–1995	-4.7∥	1995+–2002	0.2	0.8∥	0.5	1.1	0.8∥	0.5	1.1	-0.5	-1.1	0.1	-1.3∥	-1.9	-0.6
Female	1975–1979	-0.2	1979+–1987	1.5∥	1987+–2002	0.3∥			0.7∥	0.6	0.8	0.6∥	0.3	0.8	0.5∥	0.1	0.9	0.3∥	0.2	0.5
Top 15 for males																				
Prostate	1975–1988	2.6∥	1988+–1992	16.5∥	1992+–1995	-11.2∥	1995+–2002	1.7∥	2.8∥	1.9	3.7	2.6∥	1.8	3.5	-0.3	-1.9	1.4	-2.4∥	-4.1	-0.6
Lung and bronchus	1975–1982	1.5∥	1982+–1991	-0.4	1991+–2002	-1.8∥			-0.7∥	-0.9	-0.4	-0.4∥	-0.7	-0.2	-1.7∥	-2.0	-1.4	-1.8∥	-2.1	-1.5
Colon and rectum	1975–1986	1.1∥	1986+–1995	-2.1∥	1995+–1998	1.0	1998+–2002	-2.5∥	-0.7∥	-1.0	-0.4	-0.5∥	-0.9	-0.1	-0.9∥	-1.6	-0.2	-1.3∥	-2.4	-0.3
Urinary bladder	1975–1987	1.0∥	1987+–1996	-0.5	1996+–2000	1.6	2000+–2002	-2.6	0.3∥	0.2	0.4	0.4	-0.1	0.8	0.3	-0.2	0.9	-0.1	-1.3	1.1
NonHodgkin lymphoma	1975–1991	4.3∥	1991+–2002	0.2					2.5∥	2.0	2.9	2.7∥	2.4	3.0	-0.2	-0.9	0.6	0.2	-0.3	0.6
Melanoma of the skin**	1975–1985	5.8∥	1985+–2002	3.8∥					4.2∥	4.0	4.5	4.6∥	4.2	5.0	3.9∥	3.1	4.6	3.8∥	3.5	4.1
Leukemia	1975–2002	0.1							0.1	0.0	0.3	0.1	0.0	0.3	0.6∥	0.0	1.1	0.1	0.0	0.3
Oral cavity and pharynx	1975–2002	-1.2∥							-1.2∥	-1.3	-1.0	-1.2∥	-1.3	-1.0	-1.7∥	-2.6	-0.9	-1.2∥	-1.3	-1.0
Kidney and renal pelvis	1975–2002	1.8∥							1.8∥	1.6	1.9	1.8∥	1.6	1.9	2.0∥	1.2	2.8	1.8∥	1.6	1.9
Stomach	1975–1988	-1.2∥	1988+–2002	-2.0∥					-1.6∥	-1.8	-1.5	-1.6∥	-1.9	-1.4	-2.2∥	-2.6	-1.7	-2.0∥	-2.4	-1.7
Pancreas	1975–1981	-1.8∥	1981+–1985	1.1	1985+–1990	-2.1	1990+–2002	0.1	-0.5∥	-0.7	-0.4	-0.6	-1.3	0.1	0.2	-0.3	0.7	0.1	-0.2	0.5
Liver and intrahepatic bile duct	1975–1984	1.7	1984+–1999	4.5∥	1999+–2002	-0.7			3.6∥	3.3	3.9	2.9∥	2.0	3.9	2.7∥	1.3	4.1	2.9∥	1.2	4.6
Brain and other nervous system	1975–1989	1.2∥	1989+–2002	-0.3					0.4∥	0.2	0.7	0.5∥	0.1	0.9	0.0	-0.6	0.7	-0.3	-0.9	0.2
Esophagus	1975–2002	0.8∥							0.8∥	0.5	1.0	0.8∥	0.5	1.0	0.9	-0.2	2.1	0.8∥	0.5	1.0
Larynx	1975–1988	-0.3	1988+–2002	-2.8∥					-1.6∥	-1.9	-1.3	-1.5∥	-1.8	-1.2	-2.8∥	-3.7	-2.0	-2.8∥	-3.2	-2.4
Top 15 for females																				
Breast	1975–1980	-0.4	1980+–1987	3.7∥	1987+–2002	0.4∥			1.3∥	1.0	1.5	1.0∥	0.6	1.5	0.6∥	0.0	1.3	0.4∥	0.2	0.6
Lung and bronchus	1975–1982	5.5∥	1982+–1990	3.5∥	1990+–1998	1.0∥	1998+–2002	-0.5	2.4∥	2.0	2.8	2.8∥	2.4	3.1	0.5	0.0	1.0	0.4	-0.1	1.0
Colon and rectum	1975–1985	0.3∥	1985+–1995	-1.8∥	1995+–1998	1.5	1998+–2002	-1.5∥	-0.8∥	-0.9	-0.6	-0.6∥	-1.0	-0.2	-0.3	-0.8	0.2	-0.7	-1.8	0.4
Corpus and uterus, NOS	1975–1979	-6.0∥	1979+–1988	-1.7∥	1988+–1997	0.7∥	1997+–2002	-0.6	-0.9∥	-1.2	-0.5	-1.5∥	-1.8	-1.2	0.1	-0.4	0.6	0.1	-0.4	0.5
NonHodgkin lymphoma	1975–1990	2.9∥	1990+–2002	1.2∥					2.1∥	1.8	2.3	2.2∥	1.9	2.4	1.0∥	0.4	1.7	1.2∥	0.8	1.6
Ovary¶	1975–1985	0.2	1985+–2002	-0.7∥					-0.4∥	-0.6	-0.3	-0.4∥	-0.6	-0.1	-0.6∥	-1.1	0.0	-0.7∥	-0.9	-0.5
Melanoma of the skin**	1975–1981	6.1∥	1981+–1993	2.2∥	1993+–2002	4.1∥			3.2∥	3.0	3.4	3.8∥	3.2	4.3	4.2∥	3.4	5.1	3.9∥	3.3	4.5
Pancreas	1975–1984	1.2∥	1984+–2002	-0.2					0.1	-0.1	0.3	0.3	0.0	0.6	0.4	-0.2	1.0	-0.2	-0.5	0.0
Thyroid	1975–1981	-1.2	1981+–1993	2.0∥	1993+–2002	5.3∥			2.7∥	2.3	3.2	2.2∥	1.4	3.1	5.6∥	4.7	6.6	5.0∥	4.1	5.8
Cervix uteri	1975–1981	-4.6∥	1981+–1997	-1.1∥	1997+–2002	-4.5∥			-1.9∥	-2.2	-1.7	-2.6∥	-3.2	-2.0	-2.8∥	-3.9	-1.6	-2.8∥	-4.0	-1.6
Leukemia	1975–2002	0.2∥							0.2∥	0.1	0.4	0.2∥	0.1	0.4	0.4	-0.4	1.3	0.2∥	0.1	0.4
Urinary bladder	1975–2002	0.2∥							0.2∥	0.1	0.4	0.2∥	0.1	0.4	0.3	-0.2	0.7	0.2∥	0.1	0.4
Kidney and renal pelvis	1975–1990	2.8∥	1990+–2002	1.6∥					2.2∥	2.0	2.4	2.3∥	1.9	2.7	1.5∥	0.8	2.2	1.6∥	1.0	2.2
Oral cavity and pharynx	1975–1980	2.5	1980+–2002	-0.9∥					-0.6∥	-0.8	-0.4	-0.2	-0.7	0.4	-1.0∥	-1.8	-0.1	-0.9∥	-1.1	-0.6
Stomach	1975–2002	-1.7∥							-1.7∥	-1.9	-1.5	-1.7∥	-1.9	-1.5	-0.6	-1.9	0.8	-1.7∥	-1.9	-1.5

Data are from the Surveillance, Epidemiology, and End Results Program's nine areas (San Francisco-Oakland SMSA, Connecticut, Atlanta-Metropolitan, Detroit-Metropolitan, Hawaii, Iowa, New Mexico, Seattle-Puget Sound, Utah).

*Using the public software, Joinpoint Regression Program, Version 3.0. April 2004, National Cancer Institute.

†The top 15 cancers were selected based on the sex-specific age-adjusted cancer incidence rate for 1992–2002 for all races combined.

‡ Segmented analyses with up to three joinpoints are based on rates per 100 000 age-adjusted to the 2000 U.S. Standard Population (19 age groups—Census pp. 25–1130).

§ Age-adjusted rates that were age-adjusted to the 2000 U.S. Standard Population (19 age groups—Census pp. 25–1130).

¶ All sites excludes myelodysplastic syndromes and borderline tumors; ovary excludes borderline tumors.

∥ cAPC, sAPC, or AAPC is statistically significantly different from zero (two-sided P<0.05).

** Age-adjusted rates for melanoma of the skin are calculated using white patients only.

When there are no transition points in the entire data series (e.g. leukemia for males and for females), the incident trend is linear on the log scale. Thus, exactly the same estimates are obtained for the cAPC, sAPC, and AAPC from the entire data series. The estimated sAPCs and AAPCs for the last 10-year period are exactly the same as those for the entire 28-year period. The estimated cAPCs for the last 10-year period, however, generally differ from the estimated cAPCs for the entire 28-year period. Moreover, comparing the cAPCs for the 10-year sub-time period and for the entire time period may lead to different statistical conclusions at the significance level of 0.05, although there are no transition points in the entire data series. For example, for the entire 28 years from 1975 to 2002, the estimated cAPC (also the sAPC and the AAPC) of male leukemia incidence rates is not statistically significantly different from 0; nevertheless, the estimated cAPC for the last 10 years (i.e. 1993–2002) indicates a statistically significant annual increase rate of 0.6 per cent. The estimated cAPC for 1993–2002 is not statistically significantly different from 0 for female stomach cancer incidence rates, in contrast the statistically significantly decreasing annual rate of 1.7 per cent during the entire period of 1975–2002.

When there is at least one transition point between 1975 and 2002 (that is, the assumption of a linearity of log incidence rates over the entire time period is not supported by the data), the cAPC tends to have narrower 95 per cent CIs than that of the AAPC, which indicates that the variance of the estimated cAPC tends to be underestimated when the linearity assumption does not hold. Furthermore, the estimated cAPCs are more likely to show a statistically significant difference from the null value than the AAPC estimates. For example, the segmented regression analysis reveals three transition points over the entire time period of 1975–2002 for male urinary bladder cancer incidence rates, and both the estimated AAPC and the estimated cAPC indicate that the male urinary bladder cancer incidence rate increases at 0.3 per cent annually, but the cAPC estimate indicates that this upward trend is statistically significantly different from zero, while the AAPC estimate does not. The AAPC estimate also indicates that during the 1975–2002 time period, the overall incidence trend for male pancreas cancer is flat (estimated AAPC = —0.6 per cent with the 95 per cent CI (—1.3 per cent, 0.1 per cent)), but the cAPC estimate shows a statistically significant downward trend.

For the example of the U.S. age-adjusted mortality rates for prostate cancer cited in the Introduction, the 10-year estimated AAPC from 1992 to 2001 is —3.2 per cent for whites and —1.5 per cent for blacks and both are statistically significantly different from zero. Thus, they suggest the possible benefits of PSA screening and treatment for both whites and blacks. The estimated relative rate of annual mortality change for whites, that is 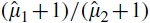, is 0.98 times that of blacks and we are 95 per cent confident that the relative rate of annual mortality change for whites versus blacks, that is (*μ*_1_ + 1)/(*μ*_2_ + 1) is somewhere between 0.975 and 0.990, where subscript 1 indicates white and subscript 2 indicates black. Therefore, the downward mortality trend in the 10 years is faster for whites than for blacks on average, which suggests that whites have derived a larger benefit from the recent cancer control advances than blacks.

Quite often in the literature, the trends were presented, rather than the data points (i.e. rates) themselves. This is a situation where the AAPC can still be applied. For instance, the trend of high school students who were current users of cigarettes from 1991 to 2005 was presented as two segmented annual per cent changes (sAPC): increasing 5.29 per cent from 1991 to 1997, and then decreasing

6.81 per cent afterwards (http://cancercontrolplanet.cancer.gov/atlas/progressreport/2007/popup.jsp?graph=both&o=j&jp=true&fig=p2&scale=auto&min=0&max=300&mt=6&t=a). We can easily calculate using AAPC, for example, the overall trend from 1991 to 2005 or the most current 10-years trend from 1996 to 2005 based on the given sAPCs.

## 4. DISCUSSION

We propose the AAPC for use in summarizing and comparing trends that may not be constant over a given time period. The proposed AAPC takes into account trend transitions, whereas the cAPC does not and thus one can be led to erroneous conclusions on statistical significance. In addition, when there are no changes in trends, (a) the AAPC reduces to the cAPC and sAPC and (b) the AAPC for any subtime intervals of specified length is exactly the same as the AAPC (and the sAPC) over the entire time interval, whereas the estimated cAPC may vary as the chosen subtime interval changes. More generally, because the estimated AAPC is based on the segmented analysis over the entire data series, any selected subinterval within a single time partition will yield the same AAPC estimate; that is it will be equal to the estimated sAPC for that time partition.

There are several specific reasons for using the AAPC instead of the cAPC and sAPC, especially in important publications that summarize national trends in cancer incidence and mortality (e.g. Howe *et al.* [14]). First, a cAPC is re-estimated over the selected subinterval, and may be sensitive to the selected subinterval; it also assumes the linearity of the trend over the subinterval. For example, in Tables 4 and 5 of Howe *et al.* [14], cAPCs are compared across cancer sites, gender, and racial/ethnic groups for the period 1995–2003. A more robust analysis would be to substitute AAPCs for the cAPCs.

Second, in Tables 2 and 3 in Howe *et al.* [14], sAPC’s are compared across cancer sites and gender. Interest often focuses on the statistical significance of the sAPC for the final segment, with the segment characterized as ‘rising’ or ‘falling’ if the final sAPC is statistically different from zero and as ‘stable’ if it is not. Comparison of these characterizations across groups may not be appropriate, however, because the standard error of an sAPC is related to the length of the segment, and final segment lengths can vary widely for different groups. For example, when 2002 was the most recent data point [15] the delay-adjusted incidence for thyroid cancer in males had an sAPC from 1980 to 2000 of 2.2 per cent, which was statistically significant, and an sAPC from 2000 to 2002 of 11.6 per cent, which was not statistically significant; the delay-adjusted incidence for thyroid cancer in females had an sAPC from 1993 to 2002 of 5.3 per cent, which was statistically significant. Thus, a characterization of the most recent segment trend for thyroid cancer in males would be ‘stable', while the characterization of the most recent segment trend for thyroid cancer in females would be ‘rising'. This comparison is not appropriate because of the radically different lengths of the final segment. To make more compatible comparisons between males and females, it is useful to compute the AAPC over the same fixed interval for both series. For example, the AAPC for 1993–2002 is 4.2 per cent for males and 5.3 per cent for females (each characterized as rising as they are both statistically significant). The AAPC for 1998–2002 is 6.8 per cent for males and 5.3 per cent for females (each characterized as rising as they are both statistically significant).

As shown in the cigarette smoking example, one additional advantage of the AAPC method is that it can be used in a situation where the trends were presented. Quite often in the literature, the trends, rather than the data points themselves, were presented.

For these reasons, we recommend using the AAPC instead of the cAPC for summarizing and comparing trends over a specified time interval. Use of the AAPC is not meant to replace sAPCs from a segmented regression analysis, because sAPCs provide a detailed picture of trends over time. When a summary trend is needed over a specified time interval, however, the AAPC provides an essential complement to the more detailed results.

We have incorporated the AAPC estimation into Joinpoint, the segmented regression analysis software program (available at http://srab.cancer.gov/joinpoint) that is used by many cancer registries throughout the world for characterizing trends in cancer rates. The software reports and compares AAPCs directly as an integral part of results from segmented regression analyses. The *Annual Report to the Nation on the Status of Cancer* and the NCI's annual publication *Cancer Statistics Review* started using the AAPC in 2008.

It is a common assumption that counts follow the Poisson distribution and the person-years denominator are fixed constants [6,16]. It is important to investigate the performance of AAPC, cAPC, and sAPC when the assumptions do not hold. This is an issue for further research.
